# Breaking down the attention-deficit/hyperactivity disorder construct to build a valid diagnosis

**DOI:** 10.1017/neu.2026.10086

**Published:** 2026-05-22

**Authors:** Kinga Szymaniak, Erica Bell, Gurubhaskar Shivakumar, Gin S. Malhi

**Affiliations:** 1 Academic Department of Psychiatry, Kolling Institute, Northern Clinical School, Faculty of Medicine and Health, The University of Sydney, Sydney, Australia; 2 CADE Clinic and Mood-T, Royal North Shore Hospital, Northern Sydney Local Health District, Sydney, Australia; 3 Adult Mental Health Unit, Hornsby and Ku-ring-gai Hospital, Northern Sydney Local Health District, Sydney, Australia; 4 Department of Psychiatry, University of Oxford, Oxford, UK; 5 Uehiro Oxford Institute, Faculty of Philosophy, University of Oxford, Oxford, UK

**Keywords:** attention-deficit/hyperactivity disorder (ADHD), diagnostic criteria, validity, reliability, DSM-5-TR (Diagnostic and Statistical Manual of Mental Disorders, Fifth Edition, Text Revision)

## Abstract

Defined by DSM-5-TR as a neurodevelopmental disorder, attention-deficit/hyperactivity disorder (ADHD) has attracted ever-mounting attention from the public, coupled with a growing interest from clinicians, researchers, and patients. This is reflected in significantly higher demand for clinical assessments and frequent media reports of a surge in ADHD cases across the lifespan. These trends are puzzling as it is unknown what they truly reflect: an improvement in clinical detection or a concerning degree of overdiagnosis? A key reason for this uncertainty is our limited understanding of the disorder and imprecision of the diagnosis – a long-running subject of criticism. To better understand these issues, in this article, we *deconstruct* ADHD through the lens of its DSM-5-TR diagnostic criteria – the basis upon which the diagnosis is routinely made. Our in-depth analysis reveals major problems associated with the diagnostic criteria with respect to their *arbitrariness*, *vagueness*, *redundancy*, and *context-dependent normality*, which together substantially undermine the validity and reliability of the diagnosis, and the ADHD construct itself, blunting the precision of ADHD research, clinical decisions, and the effectiveness of treatment – all of which are contingent on having a robust diagnosis in the first place. Hence, our detailed *deconstruction* of the diagnosis of ADHD is critical as it provides the necessary groundwork for its accurate *reconstruction* – an essential step towards developing a valid, reliable, and clinically meaningful diagnostic foundation that will inform research and improve clinical care for patients with attentional and hyperactivity–impulsivity problems.

## Graphical abstract

**Figure:**
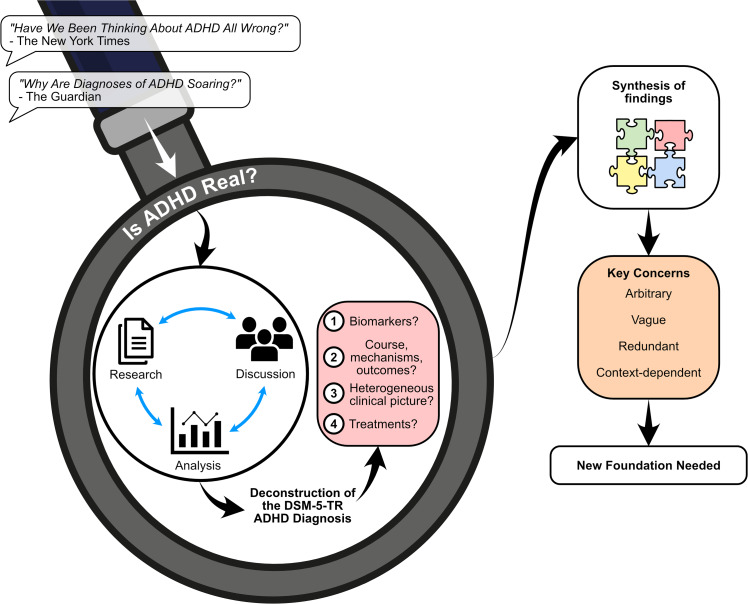

Significant outcomes

- The diagnostic criteria for ADHD are fundamentally flawed, and this leads to its overdiagnosis.
- DSM-5-TR criteria for ADHD are arbitrary, vague, and redundant, and the symptoms overlap with normality.
- The diagnosis of ADHD is not fit for purpose and must be revised to ensure accurate detection and appropriate management.

Limitations

- ADHD is an example of the crude application of DSM-5-TR criteria to the complex phenomenology of the mind.
- The diagnostic validity of ADHD remains unfounded, and in practice, it contributes to the heterogeneous clinical picture of the disorder that makes it difficult for clinicians to differentiate those who have ADHD from those who do not.
- The imprecision of ADHD diagnosis and difficulties in separating it from other psychiatric and psychological conditions mean that a significant proportion of patients are commenced on a treatment pathway that is unnecessary and potentially harmful.

*If we attend continually and promptly to the little that we can do, we shall ere long be surprised to find how little remains that we cannot do.*

Samuel Butler,

*The Note-Books of Samuel Butler* (1912)

## Introduction

The construct of attention-deficit/hyperactivity disorder (ADHD) is no stranger to criticism, with extensive commentary and challenges to its validity from a variety of conceptual, biomedical, and socio-cultural perspectives (Barkley, 2001; Visser and Jehan, 2009; Timimi and Leo, 2017; Honkasilta and Koutsoklenis, 2022; Ophir, 2024). However, recently, the disorder has attracted broader attention because of an increasing clinical demand for its formal assessment, alongside frequent media reports that claim a surge in cases, especially in adults. This has led many, including ourselves, to wonder whether the putative rise of ADHD diagnosis reflects improved detection or, alternatively, it is a consequence of overdiagnosis (Abdelnour *et al*., 2022). Notably, a recent systematic review that assessed the global prevalence of ADHD in children and adults since 2020 found no support for a definitive upward trend (Martin *et al*., 2025). Nevertheless, doubts about the clinical diagnosis of ADHD persist and have been reported in high-profile media articles, such as the recent *The New York Times* article, titled ‘Have We Been Thinking About A.D.H.D. All Wrong?’ (Tough, 2025).

In addition, we are equally baffled because of our clinical observations that are in keeping with those of our colleagues and which are supported by a careful review of recent literature that suggests as many as two-thirds of children and adolescents (66.5%) in receipt of an ADHD diagnosis also have a psychiatric comorbidity (Njardvik *et al*., 2025). As a team of clinical researchers with neuroscientific, psychology, and psychiatry expertise in mood disorders, we have been surprised by the remarkably high co-occurrence of ADHD in our patients who mainly have a primary diagnosis of depression or bipolar disorder. This high rate of comorbidity is in keeping with reports that suggest patients with mood disorders are approximately three times more likely to have ADHD than those who do not have mood disorders (Sandstrom *et al*., 2021). But, whether this is indeed the case, and more importantly, if so – *why*, remains unclear, and therefore we have turned our attention to understanding ADHD as a clinical entity.

### Preliminary remarks

Despite widespread interest in ADHD, stringent diagnosis of the disorder is still challenging for clinicians due to the lack of clarity around processes and mechanisms involved in its development, trajectory, and consequences, and together, these unknowns impose a significant risk of compromising patient care. Further, an unreliable and invalid DSM-based diagnosis is likely to hinder meaningful research.

Some might counter this view and argue that in this regard ADHD is no different from other psychiatric conditions, but we disagree, because while it is true that many DSM-based disorders suffer from diagnostic imprecision and lack sufficient scientific validity, a critique we have prosecuted ourselves (Malhi *et al*., 2020; Shivakumar *et al*., 2026), ADHD is a clear outlier. This is evident in the ongoing debate concerning overdiagnosis and mistreatment (Cortese *et al*., 2026; Szymaniak *et al*., 2026), in which the validity of ADHD as a medical condition has been called into question (Ophir, 2022). The same cannot be said about depression and anxiety, probably because their foundation is more established both in terms of clinical phenomenology and scientific evidence. For example, neuroendocrine, neuropsychological, and neuroimaging changes reliably differentiate depressed patients (Malhi and Mann, 2018; Schmaal *et al*., 2020), whereas there is no similar consensus concerning ADHD – an issue we return to later. Moreover, the core symptoms of depression and anxiety are significantly more salient than inattention. For example, it is much easier to identify uncontrollable worry or establish that a person has anhedonia or suicidal thinking and differentiate these from normality than it is to identify significant variations in attention. This is because attention is a more central brain function than mood and is geared seemingly dimensionally. In other words, while uncontrolled worrying thoughts and low mood are intermittent, attention is necessarily continuous and always *switched on*. This makes the task of clinically delimiting the boundaries of *normal* and *abnormal* attention extremely difficult, and what’s more, seriously challenges the current conceptualisation of the disorder as a discrete diagnostic category. Further, since attention is a requisite for external and internal surveillance and attention is needed to attend to oneself – this makes the empirical appraisal of attention inherently difficult.

*Our approach:* To make sense of the confusion and uncertainty surrounding the nature of ADHD, we first *deconstruc*t its DSM-5-TR diagnosis to reveal the fundamental problems from where some of the clinical difficulties emerge. This step is critical as it provides new groundwork for the reconstruction of ADHD having developed more valid and reliable criteria. Using a two-stage approach, we aim to make a novel contribution to the current debate that will facilitate a deeper understanding of ADHD.

For clarity and ease of assimilation, we have captured our thoughts in two separate but linked articles to address the two complementary parts of our approach. Note, we have chosen to focus mainly on ADHD phenomenology as this is primarily the basis for the diagnosis. Further, as we will later discuss, much of the research that lays claim to possible biomarkers, for instance, is tentative at best, and this is largely because as we have shown below the diagnosis is flawed.

In this **first article**, we have deconstructed the concept of ADHD by carefully analysing the diagnostic criteria employed by DSM-5-TR (American Psychiatric Association, 2022). This has allowed us to use the DSM diagnosis as a lens through which we can evaluate the validity of ADHD as a clinical condition. This reflects how DSM-based criteria are used to define and diagnose ADHD worldwide, both in clinical practice and in research, and are regarded as the universally endorsed standard – even though this is not the explicit intention of DSM taxonomy.

We have grounded our analysis in the combination of phenomenological and conceptual approaches: first, to examine the clinical foundation of the illness; second, to uncover how useful this foundation is in capturing key concepts on which the disorder is constructed; and third, to avoid confounding the process of making a diagnosis with aspects that are ultimately contingent on this process in the first place, such as treatment. For example, being prescribed ADHD medication is sometimes used as a diagnostic confirmation (Berk, 2025). It is precisely this type of flaw that can occur with circular thinking that we wish to avoid, and hence our focus on clinical symptomatology. At this juncture, it is important for us to invariably emphasise that our critical scientific stance towards ADHD diagnosis is not intended in any way to negate the distress that patients experience.

### Attention-deficit/hyperactivity disorder: ‘A confusing mess’ that needs tidying up?

Our concerns regarding the diagnostic validity and reliability of ADHD diagnosis are neither unique nor are they new. Initial reports of a phenomenon closely resembling ADHD date back to the end of the 18^th^ century (Crichton, 1798; Lange *et al*., 2010), and many have questioned the diagnosis since. More recent concerns go back more than a decade with, for example, Kildea *et al*. (2011), describing ADHD as ‘a confusing mess’ that professionals have difficulty understanding. This confusion is largely a consequence of gaps in our scientific and clinical knowledge about the features that supposedly uniquely characterise the disorder.

Consequently, there are several key questions concerning ADHD that warrant further investigation. For instance, ADHD is traditionally thought to be more prevalent among males compared to females, but a recent comprehensive review does not fully support this claim and instead points to the need for further research to ‘clarify [also] the nature of sex differences in ADHD’ (Babinski, 2024). Further, research into ADHD suggests that symptoms of the disorder are associated with psychological and environmental factors, such as emotion dysregulation (Soler-Gutiérrez *et al*., 2023), parental maltreatment in childhood, the relationship status of parents, and the extent to which children are exposed to media (Claussen *et al*., 2024). But whether these purported biological, psychological, and environmental associations are causal in nature, whether they exacerbate symptoms of ADHD, or indeed are the consequences of ADHD-related functional impairment is completely unknown. Once again – a key reason as to why we are unable to gain sufficient understanding about the character of these associations is that we lack a valid and reliable ADHD diagnosis that captures the psychiatric condition in a way that is meaningful and clinically useful.

## Deconstructing the phenomenon of attention-deficit/hyperactivity disorder

We begin by systematically *deconstructing* the DSM-5-TR criteria used to diagnose ADHD by examining their meaning and assessing their clinical utility. This process affords us an opportunity to include our insights into existing conceptual limitations of the diagnosis and synthesise these with our novel arguments.

### Overview of the structure of attention-deficit/hyperactivity disorder DSM-5-TR diagnostic criteria

**Figure 1.**
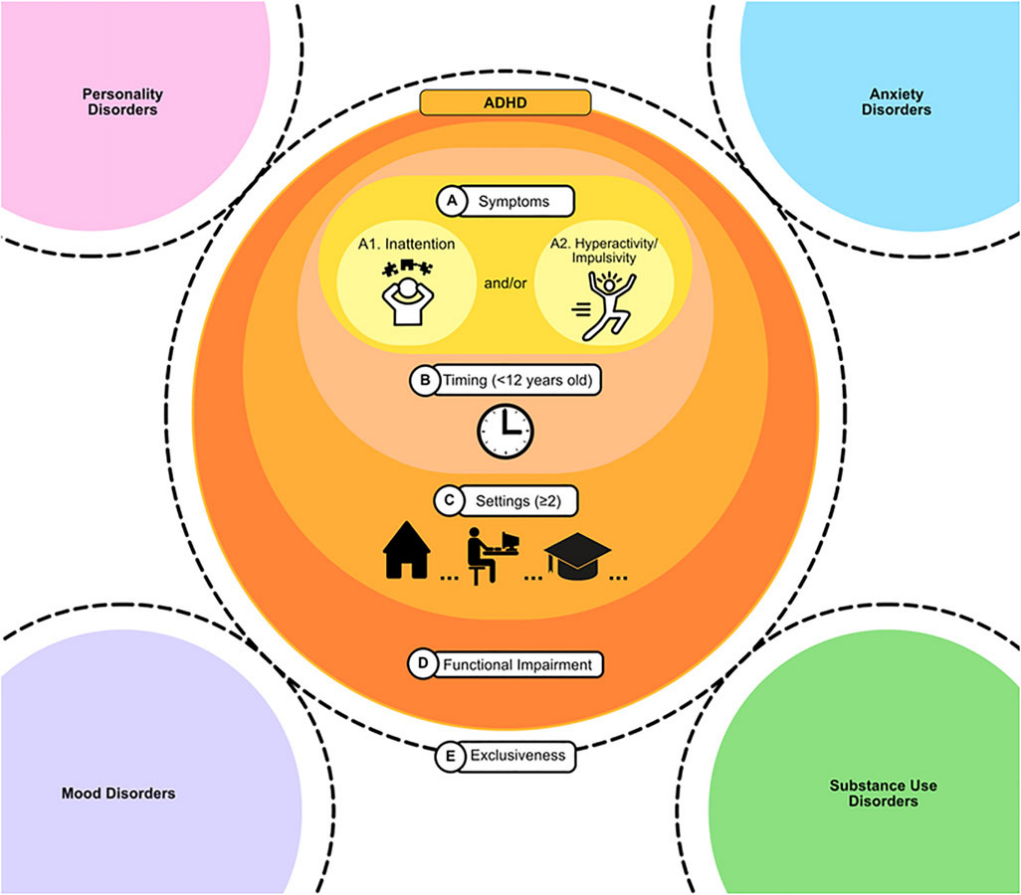
Figure 1 long description. Structuring of DSM-5-TR ADHD diagnostic criteria. The figure illustrates the diagnostic criteria (white lozenges within the central circle) upon which a DSM-5-TR ADHD diagnosis is based. **Criterion A** encompasses diagnostic symptoms and requires that a persistent pattern of inattention (A1) and/or hyperactivity–impulsivity (A2) symptoms has been observed for at least 6 months, and that this significantly interferes with a person’s development and/or functioning. **Criterion B** addresses the timing of illness onset and requires that several symptoms have been present prior to the age of 12. **Criterion C** addresses settings and indicates that several symptoms must be observed in at least two of them. **Criterion D** requires clear evidence of symptoms causing functional impairment. **Criterion E** addresses exclusiveness of the clinical picture, noting that ADHD symptoms should be distinct from other psychiatric disorders. In this figure, we have included some of the disorder groups depicted as circles in each corner of the figure that are especially at risk of being confused with ADHD due to shared symptoms. These groups include mood disorders, substance use disorders, personality disorders, and anxiety disorders.

ADHD is constructed around five diagnostic criteria, classified by DSM-5-TR as Criterion A, B, C, D, and E (see Figure 1). Each criterion addresses a different aspect of diagnosis, specifically: the symptoms (Criterion A), when (Criterion B) and where they occur (Criterion C), how functional impairment caused by the symptoms manifests (Criterion D), and finally, whether the symptoms present a clinical picture that is exclusive (Criterion E).

#### Criterion A: symptoms

**Criterion A** requires a patient to present with ‘a persistent pattern of inattention and/or hyperactivity–impulsivity’ that causes interference with either their functioning or development. DSM-5-TR considers this persistent pattern to be ‘the essential feature’ of the disorder, and it includes 18 symptoms which are divided in two separate sets. The first set (9 symptoms) is categorised under ‘inattention’ (**Criterion A1**), and the second set (9 symptoms) is categorised under ‘hyperactivity and impulsivity’ (**Criterion A2**). Depending on how many symptoms are observed per Criterion A1 and A2, the clinician diagnoses one of three presentations: (a) *combined*, (b) *predominantly inattentive*, or (c) *predominantly hyperactive/impulsive.*

**Figure 2.**
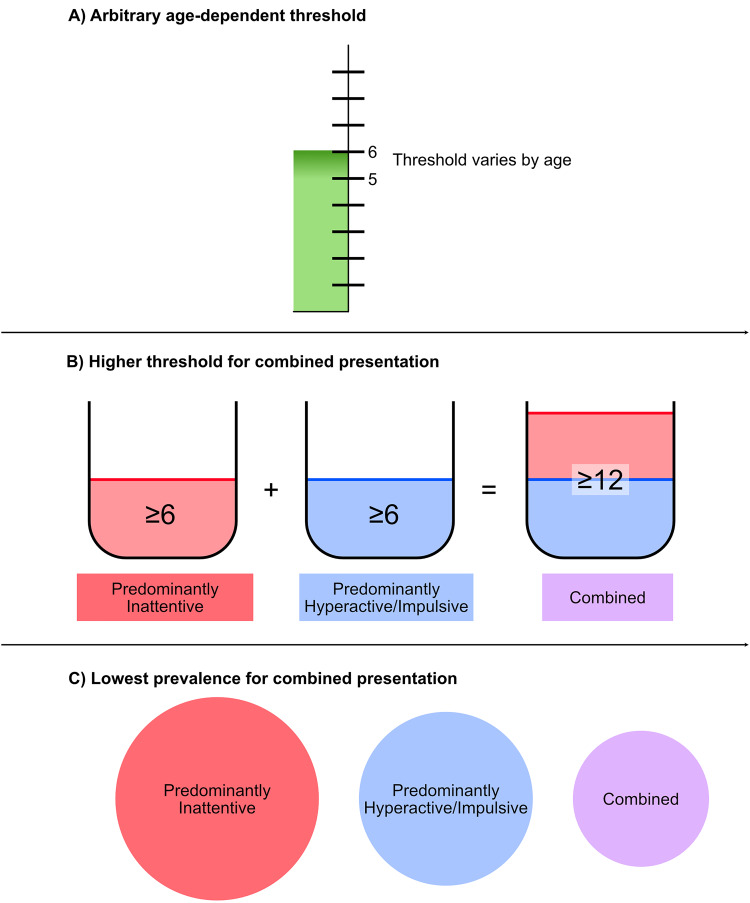
Figure 2 long description. ADHD thresholds and prevalence. DSM-5-TR differentiates three presentations (previously ‘subtypes’) of ADHD: predominantly inattentive (red), hyperactive/impulsive (blue), or combined (purple). Diagnosis of each presentation depends on whether Criterion A1, A2, or both are met (for more details about these criteria, see Figure 1 and section *Overview of the structure of attention-deficit/hyperactivity disorder DSM-5-TR diagnostic criteria*). If the required number of symptoms is observed only for Criterion A1 or Criterion A2, then a predominantly inattentive or hyperactive/impulsive presentation is diagnosed, respectively. However, if a patient manifests the required number of symptoms for both Criteria A1 and A2, then a diagnosis of a combined presentation is made. We consider this solution to be problematic because: **(A)** The number of symptoms required for Criteria A1 and A2 is age-dependent and arbitrary. Specifically, if the person is younger than 17 years, at least 6 symptoms are required, but if they are 17 years or older, this number is reduced to 5 symptoms. This change to the threshold depending on age is not informed by systematic research. In addition, though symptoms appear to differ in terms of the principal processes they capture, both within and between Criteria A1 and A2, they are given the same importance for the diagnosis and there is no hierarchy assigned to them, which directly undermines the validity of the various ADHD presentations (for more details regarding the principal processes captured by the symptoms, see Table 1 and the *Deconstructing attention-deficit/hyperactivity disorder symptoms of inattention and hyperactivity-impulsiveness* section). **(B)** Although theoretically *equal*, in practice, the clinical picture of each presentation is likely to be substantially different from the others, with the combined variant presumably entailing the most severe functional impairment. This is because it requires twice as many symptoms as the two other presentations. **(C)** The higher threshold for the combined variant explains why globally it is the least frequently diagnosed in the general adult population (Ayano *et al*., 2023). Despite these striking discrepancies, both at the phenomenological and practical levels, the pharmacological management of all three presentations is the same.

However, the number of symptoms required for each presentation is inconsistent. For instance, a *combined* ADHD presentation is diagnosed when at least 6 inattentive and at least 6 hyperactive–impulsive symptoms have been present for the past 6 months ‘to a degree that is inconsistent with developmental level and that negatively impacts directly on social and academic/occupational activities’. Thus, in total, this amounts to at least 12 symptoms, which is twice as many as required for the two other presentations (we elaborate on this issue in the legend of Figure 2). But, if the threshold of 6 symptoms is met only for Criterion A1 or A2, then either a *predominantly inattentive* or *predominantly hyperactive/impulsive* ADHD presentation is diagnosed. Further, the threshold of 6 symptoms applies only to children and adolescents younger than 17 years. For those aged 17 and older, the symptom threshold is reduced to at least 5 symptoms (see Figure 2).

**Table 1.** ADHD diagnostic criteria adapted from DSM-5-TR* (American Psychiatric Association, 2022) Table 1 long description.

ADHD symptom		Principal process
**Inattention (IA)** ***min. 6 criteria (min. 5 for 17 years old and older) for min. 6 months in min. 2 settings***		
IA-1	**Often** fails to give close attention to details or makes **careless** mistakes in schoolwork, at work, or during other activities.	Attention focus
IA-2	**Often** has **difficulty** sustaining attention in tasks or play activities.	Attention sustainability (Requires focus)
IA-3	**Often** does not **seem** to listen when spoken to directly.	Switching attention (Requires focus and sustainability)
IA-4	**Often** does not follow through on instructions and fails to finish schoolwork, chores, or duties in the workplace.	Long-term attention sustainability (Requires focus)
IA-5	**Often** has **difficulty** organising tasks and activities.	Planning and organising (Requires focus, sustainability, and switching)
IA-6	**Often** avoids, dislikes, or is reluctant to engage in tasks that require sustained mental effort.	Emotional and motivational reactions
IA-7	**Often** loses things necessary for tasks and activities.	Memory (Requires focus, sustainability, and switching)
IA-8	Is **often easily** distracted by extraneous stimuli.	Switching attention (Requires focus and sustainability)
IA-9	Is **often** forgetful in daily activities.	Memory (Requires focus, sustainability, and switching)
**Hyperactivity and impulsivity (HI)** ***min. 6 criteria (min. 5 for 17 years old and older) for min. 6 months in min. 2 settings***		
HI-1	**Often** fidgets with or taps hands or feet, or squirms in seat.	Secondary consequences of inattention
HI-2	**Often** leaves seat in situations where remaining seated is expected.	
HI-3	**Often** runs about or climbs in situations where it is **inappropriate**.	
HI-4	**Often** unable to play or engage in leisure activities **quietly**.	
HI-5	Is **often** ‘on the go’, acting as if ‘driven by a motor’.	
HI-6	**Often** talks **extensively**.	
HI-7	**Often** blurts out an answer before a question has been completed.	
HI-8	**Often** has **difficulty** waiting for their turn.	
HI-9	**Often** interrupts or intrudes on others.	

**Bold** indicates examples of semantic **vagueness**, and ***bold***, ***italic*** indicates ***arbitrariness***. What further adds to confusion are absolute conjunctions that impose presence of only one or both behaviours or contexts, while in practice, such requirements are illogical. For instance, Criterion IA-4 uses conjunction ‘and’ which indicates that a person must present with both (a) not following through instructions and (b) failing to finish a specific activity. Both (a) and (b) are two separate behaviours that can occur independently. According to the current wording both elements must be satisfied and if a person exhibits just one behaviour, they should not meet this criterion. Moreover, if (a) and (b) occur in more than one setting, e.g., school, housework, and work, the criterion should also be considered as not met. This is because conjunction ‘or’ in this criterion indicates that only one setting should be applicable if ADHD is suspected.

The right column shows the putative principal cognitive, emotional, and/or motivational processes that can be inferred from the semantic content of each inattention symptom. Hyperactivity–impulsivity symptoms, on the other hand, are all secondary to inattention given that they are contingent on difficulties in focusing, maintaining, or switching attention.

*DSM-5-TR includes examples for each listed symptom, which have been omitted in this article for brevity.

#### Criterion B: timing

**Criterion B** requires that the symptoms of inattention and/or hyperactivity–impulsivity occur before the age of 12, and for consistency of terminology, we refer to this requirement as *early age-of-onset*. Confusingly, the specific number of symptoms required to satisfy Criterion B is not provided and is described as ‘several’.

#### Criterion C: settings

**Criterion C** indicates that the symptoms of inattention and/or hyperactivity–impulsivity are present in at least two settings, for example, at home and at school. Similar to Criterion B, the non-specific term ‘several’ is used to describe the number of symptoms required to satisfy Criterion C.

#### Criterion D: functional impairment

**Criterion D** indicates that symptoms ‘interfere with, or reduce the quality of, social, academic, or occupational functioning’. Hence, this criterion appears to be secondary to Criteria A, B, and C as it refers to their consequences, namely, the functional impairment brought about by having these symptoms.

#### Criterion E: exclusiveness

Finally, **Criterion E** specifies that the symptoms of inattention and/or hyperactivity–impulsivity must not occur alongside psychotic disorders including schizophrenia and should not be better explained by any other psychiatric condition.

### General concerns regarding diagnosis

Prior to closely analysing the criteria for ADHD diagnosis, there are some general concepts upon which this diagnosis depends that are common to many psychiatric disorders, and so we address these first. Breaking down the DSM-5-TR definition of ADHD into its main components, there are two general terms about which our understanding remains limited: (a) neurodevelopmental and (b) onset.

- The introduction to *Neurodevelopmental Disorders* (Section II, DSM-5-TR) defines the term **neurodevelopmental** disorder as a set of ‘conditions with an onset in the developmental period’, wherein ‘the [neurodevelopmental] disorders typically manifest early in development, often before the child enters school’. In our view, this definition has several limitations which, to the best of our knowledge, have not been discussed previously.

**Figure 3.**
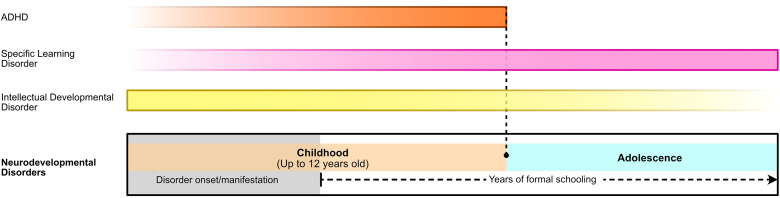
Figure 3 long description. Inconsistency of definitions for the developmental period across various DSM-5-TR neurodevelopmental disorders. A key difficulty in understanding the neurodevelopmental basis of ADHD stems from the imprecise definition of key terms, such as *developmental period* – the time when the disorder is thought to have its onset. However, the term *onset* itself is also poorly defined and interchangeably used with *manifestation*. It is also not clear when onset/manifestation refer to pathophysiology, clinically observable symptoms, or functional impairment. What adds to this confusion are the various definitions of the developmental period proposed in the manual. In the DSM-5-TR section discussing neurodevelopmental disorders, the developmental period is associated with the *pre-school* period, however later, in the description of Intellectual Developmental Disorder, its scope is broadened to encompass both childhood and adolescence, with the most severe cases likely to be detected within the first years of life. In contrast, the term *developmental period* as used in Specific Learning Disorder overlaps with years of formal schooling, with the symptoms most likely to fully manifest in later stages of school education. Importantly, each of these three descriptions of the developmental period do not match what is proposed in the description for ADHD diagnosis. Specifically, in the case of ADHD, the developmental period covers a time that only partially overlaps with other mentioned definitions, but – puzzlingly, is the only period with a strict endpoint – 12 years of age, and within ADHD diagnosis, this age demarcates the end of childhood. According to DSM-5-TR, symptoms are unlikely to be distinguishable from normal behaviour before the age of 4, being most often detectable in the elementary school period. These discrepancies across definitions, to the best of our knowledge, have not been examined, and in practice, it makes definition of the developmental period and the onset of ADHD diagnosis problematic.

First, the age at which children start school varies depending on geography, and this is not accounted for. Second, the definition suggests that DSM-5-TR considers *pre-school* to be a key part of the developmental period in every disorder categorised as *neurodevelopmental*. This is problematic as a diagnosis of ADHD requires onset to occur within the first 12 years of life, that is, many years after children have usually started school. Moreover, a *developmental period* is also inconsistently defined across other neurodevelopmental disorders. For instance, diagnostic criteria for Specific Learning Disorder describe the developmental period as ‘years of formal schooling’, while the diagnosis of Intellectual Developmental Disorder refers to it as a ‘[a period of] childhood and adolescence’ (see Figure 3). *Developmental period* as a term also appears to be variably used more broadly, outside of the manual, to indicate a range of developmental stages in the literature such as for instance adolescence (Jaworska and MacQueen, 2015) or puberty (Schneider, 2008). This conceptual heterogeneity in combination with the lack of a clear explanation as to how *the* developmental period for ADHD is precisely defined, means that it is open to interpretation.

**Figure 4.**
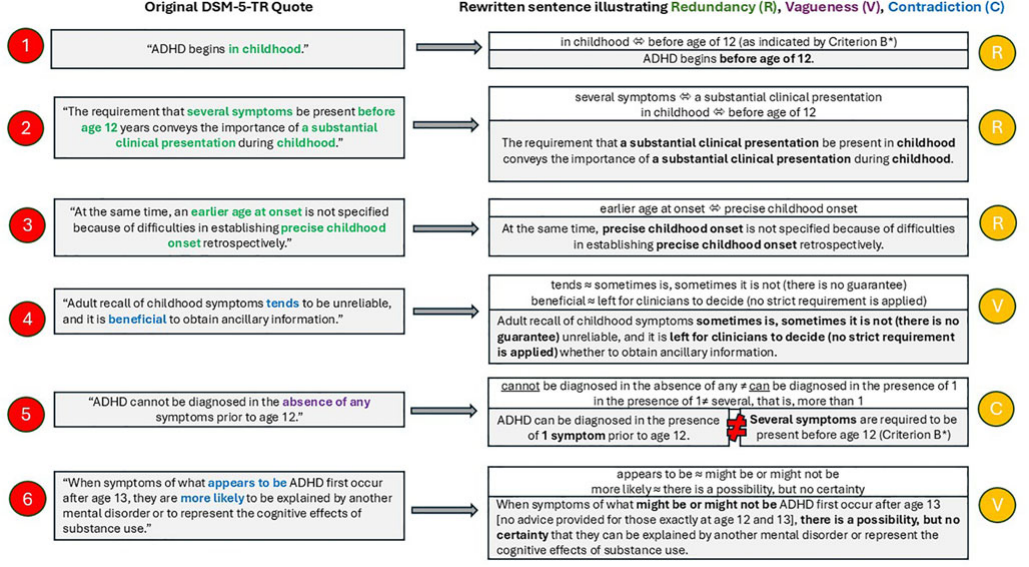
Figure 4 long description. Redundant, vague, and contradictory description of the childhood onset in DSM-5-TR ADHD diagnosis. The left column quotes the paragraph describing childhood onset under the ‘Diagnostic features’ section of ADHD DSM-5-TR diagnosis. These are provided sentence by sentence (for ease of reading, in-text citations have been removed) and words and phrases which are redundant (green), vague (blue), or cause the sentence to be in contradiction (purple) with a specific diagnostic criterion are highlighted. The right column rephrases six original sentences from the left column to illustrate that they are difficult to follow, and their exact meaning is hard to capture. Each box in the right column includes two cells. The top white cell proposes an alternative phrase to those within the original quote that cause it to lack precision or logic. The bottom grey cell rephrases the whole quote to illustrate the inherent problems. In the white cells, semantic limitations are explained by (a) listing redundant phrases next to each other (‘⬄’), (b) juxtaposing the vague phrases with their reworded version that emphasises imprecision (‘≈’) or (c) by juxtaposing statements that imply contradictory advice to the diagnostic criterion (‘≠’). Contradiction is particularly evident in quote 5, which states that ADHD cannot be diagnosed in the ‘absence’ of ‘any’ symptoms prior to age 12. Both ‘absence’ and ‘any’ are absolutes, which exclude the possibility of diagnosing ADHD when *zero* symptoms are observed in childhood. Reversing this statement, it suggests that one symptom is sufficient to consider diagnosis, which is, however, directly in contradiction with Criterion B* that requires ‘several’, that is more than one and in common parlance *more than two*, symptoms to occur prior to the age of 12. This granular analysis exemplifies the imprecision and inconsistency of the description of childhood onset that severely impacts understanding of the early age-of-onset criterion and undermines its legitimacy as a diagnostic criterion.

1. **Early age-of-onset** is specified as a key ADHD diagnostic criterion, but in practice, this is difficult to pinpoint. Unlike biological markers that allow physicians to determine with reasonable confidence the onset of medical diseases, in psychiatry, when most disorders first appear is often unclear. Further, the DSM-5-TR description of early age-of-onset is unhelpful as the criteria are vague and contradictory and show needless redundancy (see Figure 4). Moreover, DSM-5-TR does not clearly differentiate between various *types* of onset and instead conflates the onset of *symptoms* with the onset of *functional impairment*, even though the two are not necessarily coterminous (Barkley and Biederman, 1997). For instance, a person might present with inattention symptoms which do not interfere or reduce their functioning because of protective factors such as a supportive environment or personal resilience, or because detectable functional impairment is delayed in relation to when symptoms first occur. Conversely, symptoms might occur long after the onset of biological changes in the brain. Hence, it is unclear which *type* of onset (i.e., symptoms, functional impairment, or biological change), or combinations of these, should be regarded as *the onset of the disorder*. This problem is particularly difficult to address at present as none of the various types of onset are defined in DSM-5-TR and therefore *onset* cannot be precisely captured. This is perhaps why, in practice, the onset of ADHD is usually a probabilistic assumption based solely on clinical observation, in other words – a best guess.

Considering these challenges, it is then puzzling why early age-of-onset is included as an essential diagnostic criterion of ADHD and carries the same level of importance as the purported disorder’s symptoms themselves. Additionally, no hierarchy of the symptoms has been provided in the criterion for ADHD. A potential explanation is that early age-of-onset directly follows from framing the disorder as ‘neurodevelopmental’ – an approach that was first adopted in DSM-5 published in 2013 and appears to be strongly endorsed since then, despite the continued lack of unique biomarkers. Before we present our own view on this issue in more detail, we will first *deconstruct* the neurodevelopmental foundation of ADHD to better understand its meaning and implications.

### Questioning the neurodevelopmental basis of attention-deficit/hyperactivity disorder

Based on the early age-of-onset requirement and the description of the course and development of ADHD in DSM-5-TR, two potential trajectories are implied. For purposes of our current analysis we accept this bifurcation and have termed the first trajectory as **early-onset stable** or **ascending** (i.e., with symptoms severity remaining relatively the same or increasing over time) based on the DSM-TR-5 description that ‘a substantial proportion of children with ADHD remain relatively impaired into adulthood’ and that ‘[ADHD] is relatively stable through early adolescence, but some individuals have a worsened course with development of antisocial behaviours’. It is also implied that for a subgroup of individuals their impairment diminishes over time (i.e., as opposed to staying impaired), and therefore, we have termed the second probable trajectory as an **early-onset descending**. Some diminution of symptoms is also implicated by the statement that ‘in adulthood, along with inattention and restlessness, impulsivity may remain problematic even when hyperactivity has diminished’. Notably, however, what the DSM-5-TR approach absolutely rules out by indicating that ‘ADHD begins in childhood’, is a trajectory with a late onset, with symptoms first manifesting in late adolescence or during adulthood (**late-onset trajectory**). Importantly, this trajectory does not necessarily exclude the possibility of a neurodevelopmental condition, as it might simply be *latent* in childhood – lying dormant until a specific trigger activates its clinical manifestation, somewhat akin to how an arteriovenous malformation leads to stroke later in life. However, if such a trajectory exists, then it has major implications both for the diagnosis and management of ADHD, neither of which currently explicitly consider different trajectory variants.

#### Early onset in attention-deficit/hyperactivity disorder neurodevelopment

One means of anchoring ADHD to childhood would be to have evidence of biological aetiology, which, as we have already touched upon, has not been sufficiently proven. Even DSM-5-TR explicitly concedes that ‘no biological marker is diagnostic for ADHD’ and adds that ‘no form of neuroimaging can be used’ for diagnosis of the disorder. These statements are valid, but, also perplexing, as they contradict the framing of ADHD as a neurodevelopmental condition. Indeed, categorising ADHD as a disorder of early brain development is only partially supported by biological research such as genetic studies. For instance, a genome-wide association study meta-analysis of 27 loci pointed to almost 80 genes associated with ADHD that were expressed in early brain development, but none of the ADHD risk variants have been confirmed as being unique to the disorder (Demontis *et al*., 2023). Yet, ADHD is still regarded as a highly heritable disorder with some studies estimating its heritability as high as 74% (Faraone and Larsson, 2019), while other researchers point to the fact that none of the genes thought to be involved in the heritability of the disorder ‘account for more than a trivial amount of variance in the manifestation of ADHD’ (Killeen, 2019).

Support for a neurodevelopmental basis of the disorder has also been inferred, to some extent, from neurobiological studies of children and adolescents. For instance, a study conducted by Shaw *et al*. (2007) has shown a delay in achieving cortical maturation in prefrontal regions of the brain associated with ADHD. Specifically, children diagnosed with the disorder had half of the cortical points of the cerebrum reaching their maximum thickness by the median age of 10.5 years – approximately three years later than a comparison group without the disorder. Another longitudinal study that investigated developmental brain changes in ADHD individuals aged between 7 and 19 (Chang *et al*., 2024) found that the baseline striatal and cerebellar volumes predicted severity of ADHD symptoms later in life. Further questioning such biological associations, a review of ADHD neuroimaging studies revealed a lack of agreement with respect to both structural and functional abnormalities in the brain thought to be associated with ADHD – ‘likely because of the heterogeneity of the disorder’, which led the authors to conclude that ‘no definitive structural or functional pattern defines the disorder from a neuroradiologic perspective’ (Firouzabadi *et al*., 2021). Nonetheless, research examining ADHD biomarkers is continuously growing, and perhaps markers with ‘encouraging potential’ (Hurjui *et al*., 2025) will be used in the future to assist in ADHD diagnosis and differentiation of the disorder from other psychiatric conditions. We agree and are hopeful, but this should only be regarded as a future possibility once sufficient research based on reliable and valid measures of ADHD has been conducted.

#### Trajectory

Moving to the issue of whether there is enough scientific support for the ADHD trajectories implicated in DSM-5-TR, this appears to be limited, with some studies contradicting the assumption of early age-of-onset as a *sine qua non* of the disorder. For instance, Breda *et al*. (2021) evaluated symptoms of ADHD longitudinally in four waves (from 11 to 22 years of age) and found that at the age of 22, the majority of individuals had a stable neurodevelopmental trajectory, with a relatively consistent pattern of symptoms since childhood. However, approximately one-fifth of the sample, amounting to 118 individuals, was classified as presenting an ascending trajectory of symptoms, defined by authors as ‘[having] high probability of late onset’. In other words, this study introduces the possibility of ADHD starting later in life, thereby providing some valuable insights as to how ADHD symptoms might evolve over time.

The early age-of-onset approach adopted by DSM-5-TR has also been challenged by a longitudinal cohort study conducted by Moffitt *et al*. (2015), who assessed ADHD symptoms in waves from birth to the age of 38. Contrary to the expectations of the researchers, and the DSM-5-TR model of the disorder, the group with ADHD diagnosed in childhood (61 individuals) and the group diagnosed in adulthood were almost completely distinct. Indeed, only three childhood ADHD cases met diagnostic criteria during the last wave – at the age of 38, constituting approximately 10% of the adult ADHD group (31 individuals). This, again, contradicts the assumption that ADHD is solely an early age-of-onset condition and implies that ADHD symptoms may also come to the fore later in life, either because of an endogenous trigger that activates a latent neurodevelopmental condition, or because there are some other external (e.g., supportive adults) or internal (e.g., child’s resilience) factors that attenuate symptoms and associated impairment (Mitchell *et al*., 2021). Based on our knowledge, however, different variants of ADHD trajectories and factors that are likely to shape them have not been sufficiently examined by longitudinal research to date, and hence we do not know which trajectories apply. Relatedly, and perhaps an even more contentious issue in the light of the literature, is the validity of stipulating an early age-of-onset based on the definition of ADHD as a neurodevelopmental condition. To our minds, there does not seem sufficient research or logic to support this specific requirement. Therefore, we now briefly review this research.

#### Early age-of-onset criterion

Implementation of a strict age-of-onset criterion has been widely criticised (Asherson *et al*., 2016; Honkasilta and Koutsoklenis, 2022; Koutsoklenis and Honkasilta, 2023), and we endorse these views, as the criterion seems arbitrary. The cut-off was increased from 7 years old in DSM-IV (American Psychiatric Association, 1994) to 12 years old in DSM-5 with no evidence-based explanation for this 5-year shift, which expanded the scope of the diagnosis considerably. Researchers who evaluated whether extending the age cut-off was justified based on existing findings concluded that ‘changes to the age of onset criterion were based on minimal research evidence that suffered from either high risk of bias or poor applicability’ (Sanders *et al*., 2019). Further, a prospective birth cohort study from the UK found that adults who reported having symptoms prior to the age of 12 had also exhibited ADHD symptoms prior to the age of 7 (Polanczyk *et al*., 2010), suggesting that there is no detectable difference between these ages.

The idea of strict early age-of-onset is also challenged by recent groundbreaking research, which has identified four turning points (around the ages of 9, 32, 66, and 83) marking five phases in the topological development of the human brain. From the study’s findings, the age of nine marks the end of the childhood phase, while the age of 32 – the end of adolescence, and then adulthood ends around the age of 66, followed by early ageing, which goes on until around 83 when late ageing starts (Mousley *et al*., 2025). It is therefore around the age of 32 when ‘the most directional changes and a large shift in trajectory occur compared to the other turning points’, and importantly, this milestone is largely ‘context-dependent rather than [reflecting] a purely biological shift’ (Mousley *et al*., 2025). What this means is that changes in brain development have a phasic nature over the lifespan, with each phase having its distinct features influenced by both environment and biology. Clearly, this research further undermines the validity of an early age-of-onset criterion, which does not reflect the ‘non-linear nature of human development’ that this research documents (Mousley *et al*., 2025). We will return to this critical issue in our next article, in which we *reconstruct* the concept of ADHD.

Other findings that cast doubt on the broader issue of whether strictly implementing early age-of-onset is a valid approach are the variations in ADHD prevalence that depend on whether the early age-of-onset criterion was considered in the ADHD diagnosis or not. For instance, a systematic review and meta-analysis of data reporting ADHD prevalence in the general population (Song *et al*., 2021) found that in 2020, the global prevalence of ADHD among adults whose diagnosis required an early age-of-onset criterion to be stringently met was 2.58%, while prevalence for those whose diagnosis did not impose this criterion was approximately three times higher (6.76%). This difference highlights the critical role the scientifically unjustified early age-of-onset criterion plays in determining whether a person receives the diagnosis of ADHD and in consequence is prescribed treatment. This clearly warrants further consideration.

#### Retrospective recall

A key reason why we argue that the early age-of-onset criterion is of doubtful validity is that it forces clinicians to base their assessment on retrospective recall, which is inherently flawed. *Prima facie*, it seems illogical to ask a person who has a purportedly neurodevelopmental-based deficit of attention to reliably recall their symptoms from years gone by, especially considering that even those without any such deficit may struggle to do so reliably. This also applies to parents who are often required to recall if their child had ADHD symptoms when younger as a part of diagnostic assessments. Their ability to recall these details concerning childhood is limited, as over time memories naturally fade which is in line with findings showing that only 23% of parents whose children were diagnosed with ADHD in childhood were able to recall either this diagnosis or occurrence of childhood ADHD symptoms twenty years later when their children reached their late 30s (Moffitt *et al*., 2015).

Retrospective recall is similarly poor in patients. For instance, researchers who conducted two assessments of ADHD symptoms (Miller *et al*., 2010), first, in early childhood, and second – in late adolescents, found that 62.8% of adolescents who met criteria for ADHD in childhood (including all three presentations: combined, inattentive and/or hyperactivity–impulsivity) were able to recall enough childhood symptoms during the follow-up assessment to justify their past ADHD diagnosis^1^. However, when this diagnostic threshold was tightened to consider solely the group that met criteria for a combined ADHD presentation – which in fact requires meeting twice as many symptoms as the other two presentations (see Figure 2) – in their childhood, nearly 70% failed to recall enough symptoms that would support their childhood ADHD diagnosis. Similarly, another study found that only 40.7% of adult ADHD cases who believed they had symptoms of ADHD in childhood recalled it correctly in their adulthood (Breda *et al*., 2020). Conversely, of those who did *not* believe that they had ADHD symptoms in childhood, 62.1% were correct. This equates to total accuracy of retrospective recall of approximately 55.4% (Breda *et al*., 2020). Of note, in this study, adult ADHD was assessed solely using DSM-5 diagnostic criteria, while excluding the early age-of-onset criterion from the analysis. Removing this criterion was justified by the authors as an ‘approach [that] emulates clinical practice, when clinicians face individuals presenting [with] a current ADHD syndrome and the only available retrospective data on childhood ADHD symptoms is the patients’ self-retrospective recall’ (Breda *et al*., 2020). This approach to classification is important because it draws attention to an important issue – another instance of circularity of the logic operating within ADHD diagnostic criteria, where the diagnosis is based on proving that the patient had symptoms prior to 12 years old. However, this is rarely attained and then forces clinicians to verify the early age-of-onset through alternative means that are more readily accessible and convenient, such as retrospective recall, even though recalling symptoms in this manner, as discussed, is proven to be at high risk of inaccuracy. Therefore, this approach severely undermines the integrity of the diagnosis.

In sum, based on the evidence to date, it is perhaps reasonable to maintain the *possibility* that ADHD is a neurodevelopmental disorder that has a biological substrate, and we subscribe to this hypothesis – although with extreme caution. This is because it is critical that this hypothesis is first scientifically verified. Until any doubts in this regard are fully eliminated, the legitimacy of framing ADHD as a neurodevelopmental condition remains questionable. Therefore, we are puzzled by the attachment of the field to this framework along with the assumption of a neurodevelopmental basis to ADHD that has rarely been challenged by scientific inquiry. We suspect that it is partly because of implicit advantages that this approach likely involves. For instance, because neurodevelopmental changes might be masked by various factors up until adulthood, using the neurodevelopmental framework keeps the scope of inquiry relatively broad and allows for scanning the spectrum of ADHD symptoms at all stages of life, which indeed can be helpful in gaining a better understanding of protective factors and trajectories of the illness. However, using this framework without a solid and complete scientific justification also carries hidden risks. Specifically, classifying ADHD as a neurodevelopmental illness creates a false impression of established credibility of the disorder and its treatments, and allows it to circumvent the need for diagnostic validity by suggesting that biomarkers unique to the disorder exist but that they merely haven’t been discovered as yet. This is problematic because it means that in practice, the purported neurodevelopmental basis of ADHD is rarely challenged nor scientifically investigated. Instead, it is simply assumed to be the case, which in turn hinders our progress of gaining a better understanding of whether ADHD is a biologically driven condition and if so, to what extent this is the case and what is its nature.

In addition, returning to trajectories, the potential variations of the course of the disorder, and whether these must necessarily involve an early age-of-onset, warrants further investigation. Alternative possibilities should also be considered, such as the existence of a late-onset ADHD variant that is neurodevelopmental in origin, but only begins to manifest later in life, following a time-sensitive trigger, or that a late-onset inattention deficit is a completely different disorder from what we currently consider to be ADHD. Since none of these alternatives neatly fits the approach proposed by DSM-5-TR, and most of the reviewed literature points to a stable or descending trajectory as the most likely common variants, we – again, cautiously – submit to the stipulative approach taken by DSM-5-TR that ADHD is likely to have a neurodevelopmental foundation that starts to develop in childhood, yet due to the fact that this foundation may not manifest until late in life, we do not subscribe to necessitating an early age-of-onset in the diagnosis. We also consider the current approach of linking the onset of the disorder to a specific age as clearly lacking validity. Similarly, we do not subscribe to the strategy of founding the ADHD diagnosis on retrospective recall as this hamstrings its reliability and validity.

Having pointed out the many limitations of ADHD diagnosis, we now raise our specific concerns as we *deconstruct* the symptoms upon which the diagnosis is based.

## Deconstructing attention-deficit/hyperactivity disorder symptoms of inattention and hyperactivity-impulsiveness

We begin our *deconstruction* of the symptoms of ADHD by examining their descriptions in the DSM-5-TR. The first point we noticed is that DSM-5-TR assigns equal weight to each symptom even though individual symptoms clearly vary substantially in terms of how closely they relate to attentional difficulties. This observation also aligns with symptom network analyses which have revealed that some symptoms play a more central role in the diagnosis than the others (Silk *et al*., 2019). This is apparent when the contribution made by each symptom to the overall diagnosis is carefully examined at face value. For example, in our view, the symptom ‘fails to give close attention to details’ (IA-1) is more closely related to attentional focus, whereas ‘has difficulty organizing’ (IA-5) relates more so to higher executive functions – planning and organising. Being ‘forgetful’ (IA-9), on the other hand, is nearer to a dysfunction of memory, rather than attention. For reference and discussion, the key ADHD symptoms adapted from DSM-5-TR are presented in Table 1 along with the processes that we suggest they appear to primarily address.

Furthermore, while the eight of the inattention symptoms refer to cognitive functioning, one symptom – ‘avoids, dislikes, or is reluctant to engage in tasks that require sustained mental effort’ (IA-6) – arguably concentrates on a distinct facet of human experience, namely, emotional reaction (dislike) alongside motivational responses (avoidance and reluctance). Another symptom that can have differing explanations is ‘does not follow through on instructions and fails to finish schoolwork’ (IA-4). It is difficult for us to appreciate the meaning of this symptom because failing to finish a task does not necessarily mean that the person was unable to understand the task or focus on it, it is indeed possible, for instance, that the task was simply too difficult, or that the number of tasks being attended to at one time was overwhelming. Overall, the inattention symptoms might be a consequence of an inherent cognitive deficit; however, they may alternatively be a consequence of other internal or external factors that are independent of individual cognitive abilities. The meanings of the symptoms can of course be clarified with careful inquiry, but the *checklist* manner in which the criteria are currently applied does not impose sophisticated exploration of each symptom that a patient endorses.

Our second point is that hyperactivity–impulsivity symptoms are, essentially, secondary to attention, as they depend on being able to ‘attend’ to a stimulus in the first place. For instance, leaving a seat unexpectedly (HI-2), being unable to play ‘quietly’ (HI-4) and ‘blurt[ing] out an answer’ (HI-7) can be, of course, manifestation of hyperactive or impulsive behaviours, but they all could also be a consequence of difficulties in focusing and maintaining attention.

This uncertainty regarding the meaning of individual symptoms prompted us to examine them more closely, and we have grouped our observations under four headings that reflect our concerns: **(a)** arbitrariness, **(b)** vagueness, **(c)** redundancy, and **(d)** context-dependent normality. It is important to note that our insights overlap and build upon the observations of others in the field (Honkasilta and Koutsoklenis, 2022). However, the line of reasoning we provide as well as our interpretations are indeed original and have been developed through numerous in-depth discussions and granular analysis. To draw attention to the identified issues and to better illustrate them, we have highlighted the imprecision of various words and phrases in the descriptions of ADHD symptoms in Table 1.

### Concern 1: arbitrariness

Our first concern is that of **arbitrariness**, which we have already touched upon when discussing the validity of the early age-of-onset criterion, which in our view is set arbitrarily at 12 years. However, this is not the only arbitrary criterion within the diagnostic criteria for ADHD (Barkley, 2001; Honkasilta and Koutsoklenis, 2022), and other examples include the thresholds used to specify various parameters such as *the number* of symptoms required (e.g., at least six), *how long* these have to be present (at least six months), and in *how many* settings they must occur (at least two) to permit a diagnosis of ADHD. We have found no clear reason as to why these particular thresholds are applied, which is all the more confusing given that they are inherently difficult to map onto the features of the disorder, and their application is not informed by research. Recent findings have challenged these cut-offs, and if we are to adopt a pragmatic stance, it is difficult to justify why a 17-year-old with 5 symptoms of inattention qualifies for the disorder but another individual of the same age with only 4 symptoms does not – especially as the symptoms are not specified. Moreover, research has shown that thresholds of 5 and 6 symptoms do not significantly differ with respect to the functional impairment they confer in presumed cases of ADHD (Hartung *et al*., 2019). Therefore, while strict thresholds regarding numbers of symptoms might at first appear to be helpful in that they provide a quantifiable measure that can be used to delineate the disorder, in the absence of scientific evidence to support these cut-offs, they risk missing genuine diagnoses, especially those that are emerging. Thus, in our view, these arbitrary cut-offs give a spurious impression of a sharp boundary that distinguishes ADHD when, in fact, the edges of the disorder are largely fuzzy.

### Concern 2: vagueness

Reviewing the diagnostic criteria for ADHD, many of the words and phrases used to describe the symptoms are informal and imprecise in their meaning, creating semantic **vagueness** (Freedman and Honkasilta, 2017; Honkasilta and Koutsoklenis, 2022). This is doubly confusing, as vague terms not only fail to specify characteristics but also compound the number of ways in which symptoms can be interpreted. In other words, symptom vagueness decreases both diagnostic validity and reliability. For instance, many ADHD symptoms (see Table 1) employ the word ‘often’ to describe symptom frequency; however, it can be interpreted in numerous ways, by both the patients themselves and the assessors; for some people, it might mean *a few times a day*, for others *a few times a week*. Another example is the use of the word ‘several’ to define the number of symptoms that need to be present before 12 years old and that need to occur in two or more settings in the six months prior to assessment. This is again confusing as the word ‘several’ can refer to any number that exceeds 1, and the meaning is not specified in the manual. In a similar vein, phrases like ‘careless mistakes’ (IA-1), ‘difficulty [sustaining attention]’ (IA-2), ‘inappropriate [situations]’ (HI-3), ‘[talks] extensively’ (HI-6) are subjective and lack the requisite specificity. This vagueness, as we have termed it, has significant implications for diagnostic validity and reliability, and like arbitrariness, it increases the variability and imprecision with which an ADHD diagnosis is made. Consequently, patients diagnosed with ADHD are an extremely heterogeneous group phenomenologically, and yet in practice, they all receive the same diagnosis (ADHD) and undergo similar treatment. For a proportion of patients, this treatment might be reasonably well-suited, but for others it may be ineffective and expose them to treatment-related risks (Malhi *et al*., 2025). This may in part explain why ‘ADHD medications are not well tolerated by everyone, [and] there is no evidence that they significantly improve outcomes that are important to individuals with ADHD such as quality of life’ (Ostinelli *et al*., 2025).

### Concern 3: redundancy

Symptom vagueness contributes to another concern, namely, **redundancy** (Honkasilta and Koutsoklenis, 2022; Peng *et al*., 2025). Symptoms that overlap with regard to the underlying phenomenon they are trying to capture are not problematic in and of themselves. However, when combined with a lack of hierarchy (a system in which symptoms are considered more important or fundamental than others), or meaningful groupings (where the symptoms are specified and not optional), they reduce the construct validity of diagnostic criteria, and hence the reliability of the diagnosis. This is because without meaningful organisation and structuring of the diagnostic criteria, redundant symptoms allow an individual to *meet criteria* for the disorder because the symptoms overlap, without necessarily capturing the full breadth and depth of purported underlying elements that constitute the disorder.

Upon close analysis, the majority of inattention symptoms can be sorted according to three principal attention processes: (1) **focusing** on a stimulus, (2) **sustaining** this focus, and (3) **switching** this focus between stimuli (see Table 1, column ‘*Principal process*’).

Beginning with the first inattention symptom – ‘fails to give close attention to details or makes careless mistakes’ (IA-1), this refers mainly to being able to *focus* on a stimulus (e.g., an object, a person, a thought). In contrast, the second inattention symptom ‘has difficulty sustaining attention in tasks or play activities’ (IA-2) refers to sustaining attention; it is contingent on the first symptom, but adds a temporal aspect, meaning that the focus of attention must be held over time. The third inattention symptom – ‘does not seem to listen when spoken to directly’ (IA-3) refers to being able to switch attention. At first glance, this seems different in nature from the first and second inattention symptoms, but logically, to be able to switch attention from stimulus A to stimulus B, a person must first be able to focus and sustain attention on stimulus A. Hence, the third inattention symptom builds on the first two symptoms. Overall, even this basic analysis illustrates some intrinsic and necessary interdependence between the three first symptoms, and taken together they reflect the processual nature of attention. While these three symptoms interrogate successive steps of attentional processes, many of the remaining inattention symptoms are similarly derived from the inability to focus, sustain, and/or switch attention, which means they add little incremental value with respect to defining the disorder. For instance, it is difficult to point to a *qualitative* difference between some of the examples of how the symptoms manifest. Specifically, it is unclear whether there is a meaningful difference between ‘difficulty keeping materials and belongings in order; messy, disorganized work; [having] poor time management; [and] fail[ing] to meet deadlines’ (IA-5) – and – ‘[being forgetful about] doing chores, running errands; for older adolescents and adults, returning calls, paying bills, keeping appointments’ (IA-9), as both examples can be interpreted as indicating an impaired ability to focus and sustain attention or switch attention back to a more necessary task at hand.

Redundancy is also evident across the symptoms within the hyperactivity–impulsivity domain. For instance, one might ‘blurt out an answer before a question has been completed’ (HI-7), which is essentially a manifestation of a person having ‘difficulty waiting for their turn’ (HI-8). Similarly, the observation that a person ‘leaves [their] seat in situations where remaining seated is expected’ (HI-2) overlaps considerably with being ‘unable to be or uncomfortable being still for [an] extended time’, a clarification which DSM-5-TR uses as an example of symptom HI-5, the only difference being that the former symptom (i.e., HI-2) is more specific.

Lastly, there is also redundancy between symptoms of hyperactivity–impulsivity and inattention, which is a consequence of the conceptual relationship between the two symptom domains, and the fundamental role of inattention. In other words, to be able to wait for one’s turn or sit when expected to remain seated, and wait until someone has finished asking their question, one must be able to attend to stimuli that convey necessary information upon which one is anticipated to act. This initial step is essential before a person is then able to address questions that they might ask themselves such as, for instance, *what is a behaviour that is expected from me?* or *what is the question that I am being asked?* Naturally, other abilities such as delaying gratification or inhibiting reactions are also crucial for preventing impulsive or hyperactive behaviour. The problem with such redundancy, akin to their vagueness, is that collectively the symptoms fail to achieve sufficient specificity. Instead, together, they cover a broad set of thoughts and behaviours that then readily overlap with both other types of disorders as well as normal ideas and actions. This leads us to another major concern about the diagnostic criteria for ADHD, namely, the boundary between normality and the diagnosis of a psychiatric condition.

### Concern 4: context-dependent normality

Our fourth concern regarding ADHD diagnostic criteria is that in many contexts, they may have a *feature* of **normality** – that is to say, the behaviours they describe essentially lie within the boundaries of what is considered to be a normal behaviour. We recognise that *normality* is a subjective term, and that normalcy is indeed a controversial construct in and of itself, and that some might regard what is *normal* through the lens of biological primacy, such that any deviation into the territory of the disorder must be marked by pathophysiology where symptoms are highly unusual in particular social or cultural contexts. Further, we acknowledge that these phenomenological approaches based on descriptive psychopathology have their own limitations, as biological conditions may not always present observable problems, or even cause discernible dysfunction, and that similarly culturally unacceptable behaviours do not necessarily cause impairment or require treatment (Bassett and Baker, 2015). Therefore, to argue that DSM-5-TR symptoms of ADHD may at times be regarded as normal, we must first agree how to define normality, and to this end, the concept of the ‘normal–disordered boundary’ proposed by Wakefield and First (2013) is helpful as it effectively combines biological and subjective approaches to normality and captures our own view as to how best to determine whether the phenomenology of the mind veers into the pathological or remains within the bounds of health. Therefore, we have deliberately used probabilistic language to emphasise that full and complete demarcation between normality and mental disorder is difficult and subject to bias. This is because, in practice, the decision of where this line resides is usually drawn based on the clinician’s perspective of the account provided by their patient, both of which are, by nature, subjective and shaped by numerous individual factors including personal attitudes, beliefs, and past experiences – not to mention social and political contexts.

Returning to determining the ‘normal–disordered boundary’, Wakefield and First (2013) suggest it needs to involve two components:

1. ‘scientific judgment that symptoms represent an internal dysfunction, that is, a failure of some psychological mechanism to perform its biologically designed function (…)’ and,
2. ‘a value judgment that the dysfunction causes harm, usually in the form of distress or impairment’.

The *first* component is particularly difficult to apply to ADHD. The reasons for this are again two-fold. First, it is hard to work out whether symptoms reflect the failure of a particular psychological mechanism as the mechanisms involved are not defined and poorly understood. In DSM-5-TR, inattention, hyperactivity, and impulsivity are conceptualised descriptively and *defined* by lists of behaviours that are assumed to be manifestations of the relevant deficits; however, the core constructs themselves have not been expounded clearly (see section *Questioning the neurodevelopmental basis of attention-deficit/hyperactivity disorder*). Second, as already pointed out previously – we still don’t know the putative biological causes of ADHD, and therefore, how can we categorise symptoms as a dysfunction if many of the so-called symptoms are essentially normal behaviours that both children and adults exhibit at some point in their life? For instance, it is not necessarily a sign of clinical problem for an adult to become forgetful, miss deadlines, or feel as if they are constantly ‘on the go’ (HI-5), especially if they live in a society where they are required to concurrently manage multiple areas of their private and professional life. Also, it might not be necessarily abnormal for a toddler to struggle to play ‘quietly’ (HI-4) or wait ‘for their turn’ (HI-8), or for an older child to talk ‘extensively’ (HI-6). These behaviours indeed can be further nuanced by additional psychosocial factors such as an inclination towards being an extrovert or having impulsive personality traits or simply being prone to feeling stressed – because of the heightened baseline anxiety, which in and of themselves are not conditions that necessarily have pathological underpinnings. The *second* component points to functional impairment as an indicator of pathology, and as mentioned before, we believe that numerous instances of patients seeking help due to attentional difficulties is the most compelling, and perhaps the only currently available evidence that pathological forms of attentional difficulties exist. However, to what extent this problem fits the DSM-5-TR description of ADHD, if at all, we don’t know. In our view, the verification of the functional impairment (addressed by Criterion D; see Figure 1) within the context of DSM-5-TR diagnosis, is from the start set up for failure as the foundation for this assessment – remaining diagnostic criteria – fail to satisfactory describe condition that will be clinically homogeneous and distinctive from both normality and other psychiatric problems. In consequence, considering that neither of the two components of differentiating normality from pathology can be reliably assessed within the realm of ADHD DSM-5-TR diagnosis, the perception of the normality of symptoms is entirely dependent on circumstances in which and by whom the symptoms are assessed.

Summing up, the seeming context-dependent normality of many of the symptoms, together with the other discussed concerns, considerably blunts the precision of the diagnostic criteria for ADHD and further challenges the reliability and validity of the diagnosis of ADHD. In practice, the problems we have identified are reflected in the heterogeneity of the clinical picture of the disorder and the high comorbidity of ADHD with other disorders.

## Deconstructing a ‘confusing mess’

**Figure 5.**
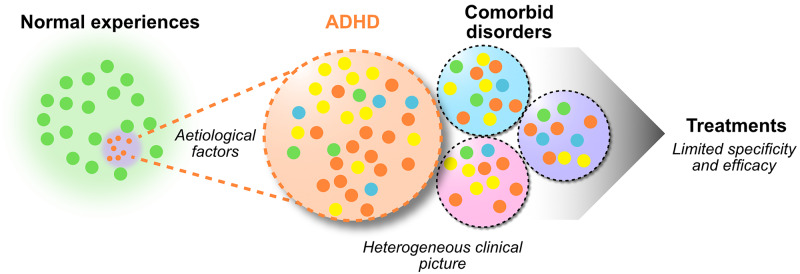
Figure 5 long description. Deconstructing ADHD clinical heterogeneity: causes and consequences. The green dots depict experiences perceived as normal. They are non-pathological in terms of aetiology and occur in the majority of people in any population. Among these experiences, there are some that are of a pathological nature (aetiological factors) that will predispose a proportion of the population to manifest behaviours perceived as abnormal, maladaptive, and/or dysfunctional. These factors include both biological and environmental aspects, and the interactions between the two. In ADHD, knowledge about these causal factors is limited, and this hinders our ability to describe the disorder in precise and accurate terms that would reflect its true nature. Consequently, symptoms which are considered to be representative of ADHD are imprecise as well, and there is no single symptom that is unique to the disorder. Indeed, literally each and every ADHD symptom can be a manifestation of another disorder or simply be a normal experience, depending on the circumstances and cultural and social context. This imprecision contributes to the heterogeneity of the clinical picture of the ADHD population and fosters high comorbidity rates. Consequently, ADHD treatments are of limited specificity and efficacy.

For more than a half a century, being able to distinguish a disorder from other disorders has been one of the key founding principles for establishing the validity of any psychiatric diagnosis (Robins and Guze, 1970), and though this criterion is seemingly addressed in the diagnosis of DSM-5-TR ADHD (Criterion E, see Figure 1), based on our current knowledge of the *disorder* and with the present means available to us, it is simply not possible to fulfil it satisfactorily. This is because, if we cannot accurately define a disorder and precisely capture its clinical presentation, we cannot confidently separate it from other diagnostic conditions. Of even greater urgency, we remain unable to distinguish patients who suffer from true attentional difficulties from those whose symptoms only deceptively mirror their problem, but in reality are of a different nature. Unpacking the potential mechanisms leading to this predicament, based on our critical analysis of the DSM-based diagnosis, it is reasonable to suggest that the high comorbidity rates between ADHD and other disorders are rooted in the limited knowledge that we have about the aetiology of ADHD as clinical entity, which in turn contributes to the imprecision of the diagnostic criteria (see Figure 5). This lack of specificity creates considerable variation within the population diagnosed with ADHD and further increases the heterogeneity of the clinical picture of the disorder, which is then difficult to distinguish from other overlapping psychiatric conditions. For instance, it is not unusual for an individual diagnosed with depression to have difficulty focusing and sustaining attention, or for patients with bipolar disorder or substance misuse to be impulsive or hyperactive. Where the boundary between these disorders and ADHD lies, and whether realistically this is even achievable in clinical practice is a matter of some conjecture. However, the practical problem here is even greater – the heterogeneity within ADHD research samples, which often utilise DSM-5-TR diagnosis of the disorder as well as the overlap of the diagnosis with other conditions, means that we lack tools that could meaningfully inform targeted interventions or could be used for the development of novel treatments (see Figure 5). Without a greater degree of specificity and utility to the ADHD diagnostic criteria, any developments in clinical guidance and intervention must therefore be treated with suspicion as to their genuine effect and relevance.

## Conclusions

In this article, the first of two that address the challenges surrounding ADHD as a clinical entity, we have *deconstructed* the disorder by examining its DSM-5-TR framework. Close analysis of the diagnostic criteria revealed major concerns, which seriously undermine the reliability and validity of the diagnosis, as they are derived from an incomplete understanding of the disorder and as such pose a significant challenge to the legitimacy of ADHD as a diagnostic category. This is in keeping with the view that ‘diagnostically speaking, [we are] standing on unstable ground’ (Studart *et al*., 2025). To this end, in the second article, we aim to revisit our concerns so as to build on them as we *reconceptualise* and *reconstruct* ADHD. Through this venture, we will offer guidance towards developing reliable and valid criteria that in conjunction with informed clinical judgement assist in making accurate evidence-based diagnosis which meets the demands of clinical and research realia. At the same time, we invite others to provide incisive and constructive criticism so that through collaborative dialogue that is grounded in scientific evidence and clinical practice, we can advance our collective knowledge.

## Footnotes

1
see *Overview of the structure of attention-deficit/hyperactivity disorder DSM-5-TR diagnostic criteria*

## Figure 1. Long description

A diagram illustrating the diagnostic criteria for ADHD according to DSM-5-TR. The central circle contains the criteria for diagnosing ADHD, including symptoms of inattention and hyperactivity-impulsivity, timing of onset before age 12, presence in more than two settings, functional impairment, and exclusiveness from other psychiatric disorders. Surrounding the central circle are additional disorder groups that may be confused with ADHD, such as mood disorders, substance use disorders, personality disorders, and anxiety disorders.

Navigate back to Figure 1..

## Figure 2. Long description

The illustration depicts ADHD thresholds and prevalence for three presentations: predominantly inattentive (red), hyperactive/impulsive (blue), and combined (purple). The first part (a) shows an arbitrary age-dependent threshold with a scale indicating that the threshold varies by age. The second part (b) illustrates that the combined presentation requires a higher threshold, combining symptoms from both predominantly inattentive and hyperactive/impulsive presentations. The third part (c) shows the lowest prevalence for the combined presentation, with circles representing the prevalence of each presentation.

Navigate back to Figure 2..

## Table 1. Long description

A table presenting ADHD diagnostic criteria adapted from DSM-5-TR. The table has two main sections: Inattention and Hyperactivity and impulsivity, each with nine criteria. Inattention criteria include details like difficulty sustaining attention, organizing tasks, and losing things. Hyperactivity and impulsivity criteria include fidgeting, leaving seats, running inappropriately, and interrupting others. Each criterion is suggested to be linked to a principal process such as, for example attention focus, sustainability or switching. The table specifies that at least six criteria must be met for individuals under 17 and at least five for those 17 and older, observed over six months in at least two settings.

Navigate back to Table 1..

## Figure 3. Long description

The line graph illustrates the inconsistency of definitions for the developmental period across various DSM-5-TR neurodevelopmental disorders. The x-axis (Neurodevelpmental disorders) represents the age range from childhood to adolescence, while the y-axis represents the different neurodevelopmental disorders. The graph includes three data lines for ADHD, Specific Learning Disorder, and Intellectual Developmental Disorder. ADHD is represented by an orange line, Specific Learning Disorder by a pink line, Intellectual Developmental Disorder by a yellow line. The graph shows that the developmental period for ADHD ends at 12 years of age, while the periods for other disorders extend beyond this age and do not have a strict age cut-off.

Navigate back to Figure 3..

## Figure 4. Long description

The table compares original DSM-5-TR quotes with rewritten sentences to highlight redundancy, vagueness, and contradiction in ADHD diagnosis criteria. It consists of six rows and two columns. The left column contains original quotes from the DSM-5-TR, each describing childhood onset in ADHD diagnosis. The right column rephrases these quotes to illustrate issues with precision and logic. Each box in the right column includes two cells: the top white cell proposes alternative phrases to those in the original quote that cause imprecision or contradiction, and the bottom grey cell rephrases the entire quote to show inherent problems. The table uses color-coding to highlight redundant (green), vague (blue), or contradictory phrases (purple). For example, quote 5 excludes the possibility of diagnosing ADHD when *zero* symptoms are observed in childhood which implies that *one* symptom is sufficient to consider diagnosisis. This, however, is in contradiction with Criterion B, which requires ‘several’ symptoms prior to age 12. This analysis underscores the imprecision and inconsistency in describing childhood onset, impacting the understanding and legitimacy of the early age-of-onset criterion.

Navigate back to Figure 4..

## Figure 5. Long description

A Venn diagram illustrating the relationship between normal experiences, ADHD, and comorbid disorders. The diagram consists of five circles which contain coloured dots that overlap between the circles. The first circle, labeled ‘Normal experiences,’ contains green dots representing non-pathological experiences common in the majority of the population, and the orange dots which underpin aetiological factors that contribute to ADHD. The second circle, labeled ‘ADHD,’ contains a mix of colored dots representing heterogeneous clinical pictures associated with ADHD. The third, fourth, and fifth circle, all together labeled ‘Comorbid disorders,’ contain dots of various colors representing different comorbid disorders. The overlapping coloured dots between the circles indicate shared experiences or symptoms among normal experiences, ADHD, and comorbid disorders. The diagram highlights the limited specificity and efficacy of ADHD treatments due to the overlap and heterogeneity of symptoms.

Navigate back to Figure 5..
